# Small G protein signaling modulator 3 (SGSM3) knockdown attenuates apoptosis and cardiogenic differentiation in rat mesenchymal stem cells exposed to hypoxia

**DOI:** 10.1371/journal.pone.0231272

**Published:** 2020-04-09

**Authors:** Seung Eun Jung, Jung-Won Choi, Hanbyeol Moon, Sena Oh, Soyeon Lim, Seahyoung Lee, Sang Woo Kim, Ki-Chul Hwang

**Affiliations:** 1 Institute for Bio-Medical Convergence, College of Medicine, Catholic Kwandong University, Gangneung-si, Gangwon-do, Republic of Korea; 2 Department of Integrated Omics for Biomedical Sciences, Graduate School, Yonsei University, Seoul, Republic of Korea; 3 International St. Mary’s Hospital, Catholic Kwandong University, Incheon Metropolitan City, Republic of Korea; Università degli Studi della Campania, ITALY

## Abstract

Connexin 43 (Cx43) may be important in cell death and survival due to cell-to-cell communication-independent mechanisms. In our previous study, we found that small G protein signaling modulator 3 (SGSM3), a partner of Cx43, contributes to myocardial infarction (MI) in rat hearts. Based on these previous results, we hypothesized that SGSM3 could also play a role in bone marrow-derived rat mesenchymal stem cells (MSCs), which differentiate into cardiomyocytes and/or cells with comparable phenotypes under low oxygen conditions. Cx43 and Cx43-related factor expression profiles were compared between normoxic and hypoxic conditions according to exposure time, and Sgsm3 gene knockdown (KD) using siRNA transfection was performed to validate the interaction between SGSM3 and Cx43 and to determine the roles of SGSM3 in rat MSCs. We identified that SGSM3 interacts with Cx43 in MSCs under different oxygen conditions and that Sgsm3 knockdown inhibits apoptosis and cardiomyocyte differentiation under hypoxic stress. SGSM3/Sgsm3 probably has an effect on MSC survival and thus therapeutic potential in diseased hearts, but SGSM3 may worsen the development of MSC-based therapeutic approaches in regenerative medicine. This study was performed to help us better understand the mechanisms involved in the therapeutic efficacy of MSCs, as well as provide data that could be used pharmacologically.

## Introduction

Mesenchymal stem cells (MSCs) can isolated various sources including bone marrow, trabecular and cortical bone, adipose tissue, skeletal muscle, peripheral blood, umbilical cord blood, and dental pulp and differentiate into multi-lineage according to sources such as osteoblast, chondrocytes, adipocytes, cardiomyocytes, tenocytes, muscle cells, fibroblast, and neuron [1–5]. Over the past decades, there has been tremendous focus on attempts to repair cardiac tissue with stem cell transplantation, and MSCs have been widely studied in both animal models and clinical trials [6,7]. MSCs are considered a promising tool with clinical implications for cell-based applications for cardiac therapeutics of myocardial infarction, peripheral ischemic vascular disease, pulmonary hypertension, and dilated cardiomyopathy [4]. Recently, signaling pathway related to some regulators containing HGF, PDGF, Wnt, and Notch-1, was found that involved in proliferation and differentiation into cardiomyocytes of MSCs [5]. In ischemic heart diseases, transplanted stem cells experience sudden oxygen deficiency when transplanted into ischemic heart tissue. Stem cells adapt themselves under hypoxic microenvironments by regulating their proliferation, differentiation, metabolic balance and other physiological processes [8,9]. The oxygen microenvironment of stem cells plays an important role in controlling stem cell properties and the ability to differentiate into different mesoderm lineages [8,9]. MSCs have practical potential for differentiation into osteogenic, chondrogenic, cardiomyogenic and adipogenic cells and/or cells with comparable phenotypes under hypoxic conditions [10–13]. These changes in the MSC response to low oxygen conditions could be used as a preconditioning method for effective stem cell transplantation. Some studies have shown that hypoxic preconditioning may promote cell survival following stem cell transplantation [14,15].

Connexin 43 (Cx43) forms intracellular communication channels and is related to cell death in impairment [16]. Lu G et al., has found that increased Cx43 expression enhances cell viability, cardiomyogenic differentiation and cardiac functions after transplantation of preconditioned MSCs [17]. Furthermore, decreases in Cx43 expression are reported for nearly every type of cardiac pathology and during the acute phase of ischemia in myocardial infarction (MI) [18–20]. Ischemic preconditioning inhibits respiratory disorder from reperfusion and mitochondrial Cx43 is closely related to these mechanisms by ischemic preconditioning [21–24]. However, the mechanism of Cx43 in myocardial protections still unknown.

Despite its short half-life (as little as 1–2 h), regulation of Cx43 appears to exist on both short- and long-term scales through protein phosphorylation and interactions and gene expression, respectively [18,20]. Although several binding partners of Cx43 with gap junction-dependent and gap junction-independent functions have been found, a study about the characterization of Cx43-binding proteins remains insufficient [25]. However, less is known about the mechanistic basis and function of Cx43 protein-protein interactions [25–28]. In our previous study, we found that small G protein signaling modulator 3 (SGSM3), a partner of Cx43, contributes to MI in rat hearts [29], and inhibiting the protective effects against oxidative stress with kenpaullone was shown to involve Cx43 and SGSM3 interactions in cardiomyocytes [30]. Based on these previous results, we expected that SGSM3 could also play a role in bone marrow-derived rat MSCs, which differentiate into cardiomyocytes and/or cells with comparable phenotypes under low oxygen conditions. Here, we determined the differential expression and interaction of Cx43 and SGSM3 in MSCs under different oxygen conditions and the effects of SGSM3 knockdown on apoptosis and cardiomyocyte differentiation under hypoxic stress. To the best of our knowledge, no studies have reported on the interaction between SGSM3 and Cx43 and their effects on damage induced by a low oxygen environment in stem cells. This study was performed to help us better understand the mechanisms involved in the therapeutic efficacy of MSCs, as well as provide data that could be used pharmacologically.

## Materials and methods

### Rat MSC culture

Second passage bone marrow-derived Sprague-Dawley (SD) rat MSCs were purchased from Cyagen (Cat. No. RASMX-01001; Santa Clara, CA, USA). The cells were cultured in Dulbecco’s modified Eagle’s medium (DMEM; HyClone, Logan, UT, USA) supplemented with 10% fetal bovine serum (FBS; HyClone, Logan, UT, USA) and 1% penicillin/streptomycin at a density of 5×10^4^ cells/cm^2^ in a 100‐mm dish in a humidified atmosphere with 5% CO_2_ at 37°C.

### Normoxic- and hypoxic-conditioned cell preparation

Rat MSCs were incubated with serum‐free media (SFM) under normoxic or hypoxic conditions for 6, 12 or 24 h. For the hypoxic conditions, cells were incubated at 37°C in 5% CO_2_, 5% H_2_ and 0.5% O_2_ in a chamber with an anaerobic atmosphere system (Technomart, Seoul, Korea). The cells were harvested after the 6‐, 12‐ or 24‐h incubation period and incubated with RIPA buffer (Cell Signaling Technology, Danvers, MA, USA) for immunoblot analysis and with TRIzol Reagent (Life Technologies, Frederick, MD, USA) for quantitative real-time RT-PCR (qRT-PCR) analysis.

### Normoxic- and hypoxic-conditioned cell preparation

Total RNA was isolated from rat MSCs under normoxic and hypoxic conditions using TRIzol Reagent, and cDNA was synthesized using a Maxime RT PreMix kit (iNtRON Biotechnology, Seongnam, Korea). The level of each gene transcript was determined quantitatively using a StepOnePlus Real-Time PCR System (Applied Biosystems, Foster City, CA, USA). A SYBR Green Dye system (SYBR Premix Ex Taq (Tli RNase Plus) with a ROX reference dye (TAKARA Bio Inc., Foster City, CA, USA) was used to perform real-time RT-PCR. All values are shown as the target gene expression level (fold change; 2ΔΔCt) normalized to the Gapdh transcript level. All primers were designed using Primer3 from BLAST (Table 1).

**Table 1:** Sequences of primers used for quantitative real-time RT-PCRs.

Genes		Primer sequence (5’– 3’)	Tm ^c)^ (°C)	Ta ^d)^ (°C)	Product length (bp)
*Genes inducible hypoxia*					
Hif1a	F ^a)^	AGCAATTCTCCAAGCCCTCC	59	60	111
	R ^b)^	TTCATCAGTGGTGGCAGTTG	57.4		
*Gap junction*					
Cx43	F	CTCACGTCCCACGGAGAAAA	59	60	119
	R	CGCGATCCTTAACGCCTTTG	59		
ZO-1	F	AGACAATAGCATCCTCCCACC	58.2	60	131
	R	TAGGGTCACAGTGTGGCAAG	58.6		
*Cx43-binding target*					
Sgsm3	F	CTGACACAGGGCAGATGAAG	57.3	60	108
	R	TCATGTGCTGTGGACGATGG	59.4		
*Internal control*					
Gapdh	F	TCTCTGCTCCTCCCTGTTCTA	58.4	60	121
	R	GGTAACCAGGCGTCCGATAC	59.3		

^a)^ F, sequence from sense strands

^b)^ R, sequence from anti-sense strands

^c)^ Tm, primer melting temperature

^d)^ Ta, primer annealing temperature

### Immunoblot analysis

Immunoblot analyses were performed as previously described [10,31]. Briefly, cell lysates were prepared with RIPA buffer containing 1% phosphatase inhibitors (Sigma‐Aldrich, St. Louis, MO, USA), 1% protease inhibitors (Sigma‐Aldrich) and 1% proteasome inhibitors (MG132; Abcam, Cambridge, UK). The proteins were separated using SDS-PAGE and transferred to polyvinylidene difluoride (PVDF; Sigma‐Aldrich) membranes. The membranes were incubated with the appropriate primary antibodies and horseradish peroxidase (HRP)‐conjugated secondary antibodies (Santa Cruz Biotechnology, Santa Cruz, CA, USA). The blots were developed with enhanced chemiluminescence (ECL Western Blotting Detection Kit, GE Healthcare, Buckinghamshire, UK), and the band intensities were quantified using ImageJ software (NIH).

### Transient Cx43 and Sgsm3 knockdown

To knockdown (KD) Hif1a and Sgsm3, target-specific commercial AccuTarget siRNAs (BIONEER, Daejeon, Korea) (Hif1a siRNA no. 1654508: sense (5΄-3΄), CAGUUACGAUUGUGAAGUU (dTdT); antisense (5΄-3΄), AACUUCACAAUCGUAACU G (dTdT); Sgsm3 siRNA no. 1752125: sense (5΄-3΄), CUGAUACAGUCGGAGAACU (dTdT); antisense (5΄-3΄), AGUUCUCCGACUGU AUCAG (dTdT)) were designed, and a negative control (nontargeting siRNA) was used. MSCs (1 × 10^6^ cells per dish in a 10-mm dish) were transiently transfected with siRNA (100 nM per dish) and agent (45 μl per dish) using the TransIT-X2 Dynamic Delivery System (Mirus Bio LLC, Madison, WI, USA), and gene expression levels were examined in the cells after cultured for 24 h. The level of each gene transcript was normalized to the level of the Gapdh transcript.

### Cell viability assay

MSCs were seeded at a density of 1×10^4^ cells/well in a 96-well plate before transfection. They were transiently transfected with SGSM3 siRNA (100 nM per dish) and agent (45 μl per dish) using the TransIT-X2 Dynamic Delivery System and exposed to hypoxic stress for 12 h after 24 h. The viability of MSCs was measured using Ez-Cytox Colorimetric Cell Viability Assay Kit (DOGEN, Seoul, Korea), following the manufacturer’s instructions. This assay is based on features of water-soluble tetrazolium salt.

### Immunofluorescence analysis

To investigate the expression patterns of cardiogenic markers upon Sgsm3 knockdown in MSCs under normoxic and hypoxic conditions, cells were grown on cell culture slides (SPL, Pocheon-si, Korea) and then fixed with 4% formaldehyde. The cells were then washed with PBS and subjected to permeabilization in 0.25% Triton X-100 (Sigma-Aldrich, St. Louis, MO, USA). The cell slides were washed with PBS three times, blocked with 1% BSA in PBS-T for 1 h, and then incubated with a monoclonal anti-cardiac troponin T antibody (1:200 dilution) and anti-GATA antibody (1:200) (abcam) overnight at 4°C. Next, the cells were washed three times with PBS. The cell slides were then incubated with a FITC-conjugated mouse secondary antibody (1:1000 dilution) against cardiac troponin T or a rhodamine-conjugated rabbit secondary antibody (1:1000 dilution) against GATA4. DAPI (Sigma-Aldrich) was used to stain the cell nuclei. The prepared slides were observed using an LSM700 confocal laser scanning microscope (Carl Zeiss, Oberkochen, Germany). Acquisition of the images was performed using Zen black or blue software (Carl Zeiss).

### Statistical analysis

All experimental results were compared using one-way analysis of variance (ANOVA) in the Statistical Package of Social Science (SPSS, version 17) program. The data are expressed as the mean ± SEM. A protected least-significant difference (LSD) test, which is a method consisting of single-step procedures in a one-way ANOVA for analyzing multiple comparisons, was used to identify significant differences between means (*p*<0.05).

## Results

### Changes in Cx43 and SGSM3 in rat MSCs under low oxygen conditions

**Fig 1:**
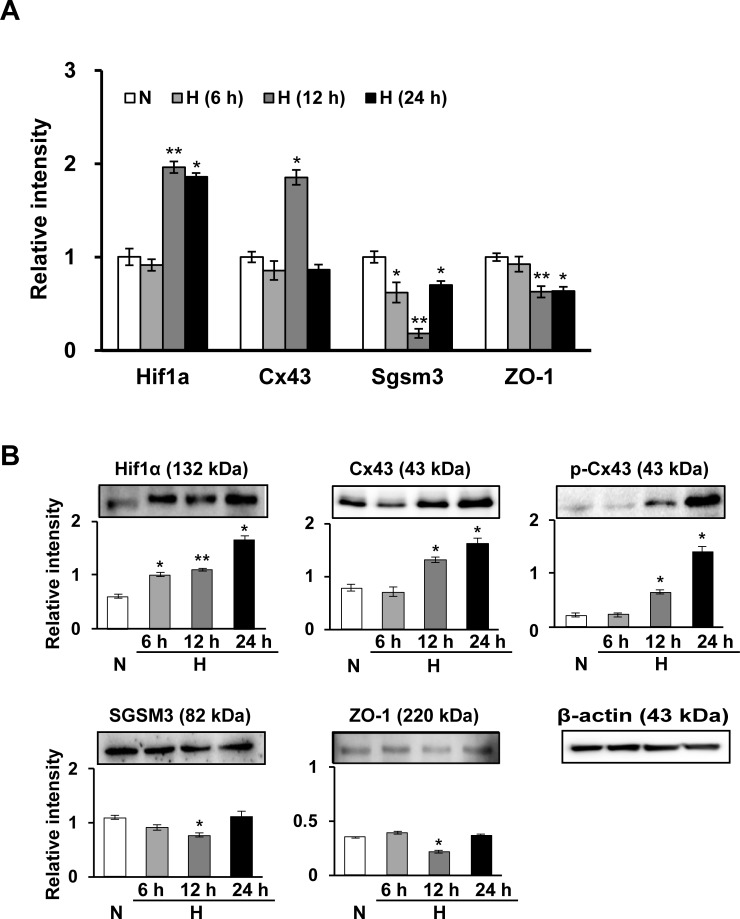
Time-dependent differential expression of gap junction factors (Cx43, ZO-1) and SGSM3 in normoxic and hypoxic conditions in rat MSCs as determined by qRT-PCR (A) and immunoblot analysis (B). All qRT-PCR values are shown as the normalized target gene expression level relative to the GAPDH transcript levels, and the data are representative of three independent experiments (A). Band intensity was measured as the area density and analyzed in ImageJ and relative intensity levels indicate protein levels normalized to the β-actin levels (B). Significant differences between the normoxia and hypoxia groups were determined via ANOVA, with *p* values indicated as *p<0.01 and **p<0.001. N, normoxia; H, hypoxia; NC, negative control cells; KD, knockdown cells.

In a previous study, we performed a coimmunoprecipitation (CoIP) assay to identify potential Cx43 partner proteins, a peptide mass fingerprinting (PMF) analysis to identify proteins interacting with Cx43 and a network analysis using GeneMANIA to validate their correlation and find additional Cx43 partner proteins. Ultimately, SGSM3, a Cx43-interacting protein, was confirmed in rat hearts [29]. Here, we carried out these three analyses in rat MSCs as preliminary experiments and showed that there was also an interaction between Cx43 and SGSM3 in MSCs. Based on these data, Cx43 and Cx43-related factor expression profiles in MSCs were first compared between normoxic and hypoxic conditions according to exposure times. Next, we investigated the gene/protein expression of Cx43, SGSM3 and tight junction protein 1 (ZO-1), and there were differences in the expression patterns of these targets between normoxic- and hypoxic-conditioned MSCs (Fig 1). Gene and/or protein levels of hypoxia-inducible factor 1-α (Hif1α) and Cx43 were significantly increased, whereas SGSM3 and ZO-1 levels were reduced by hypoxic stress; however, the hypoxia time with the most or least expression was different for gene and protein expression (Fig 1). Accordingly, we decided to harvest cells 12 h after hypoxic stress because the conditions had distinct and differential expression patterns between the normoxic and hypoxic conditions (Fig 1).

### Effects of Hif1a and Sgsm3 knockdown on the expression levels of gap junction targets

**Fig 2:**
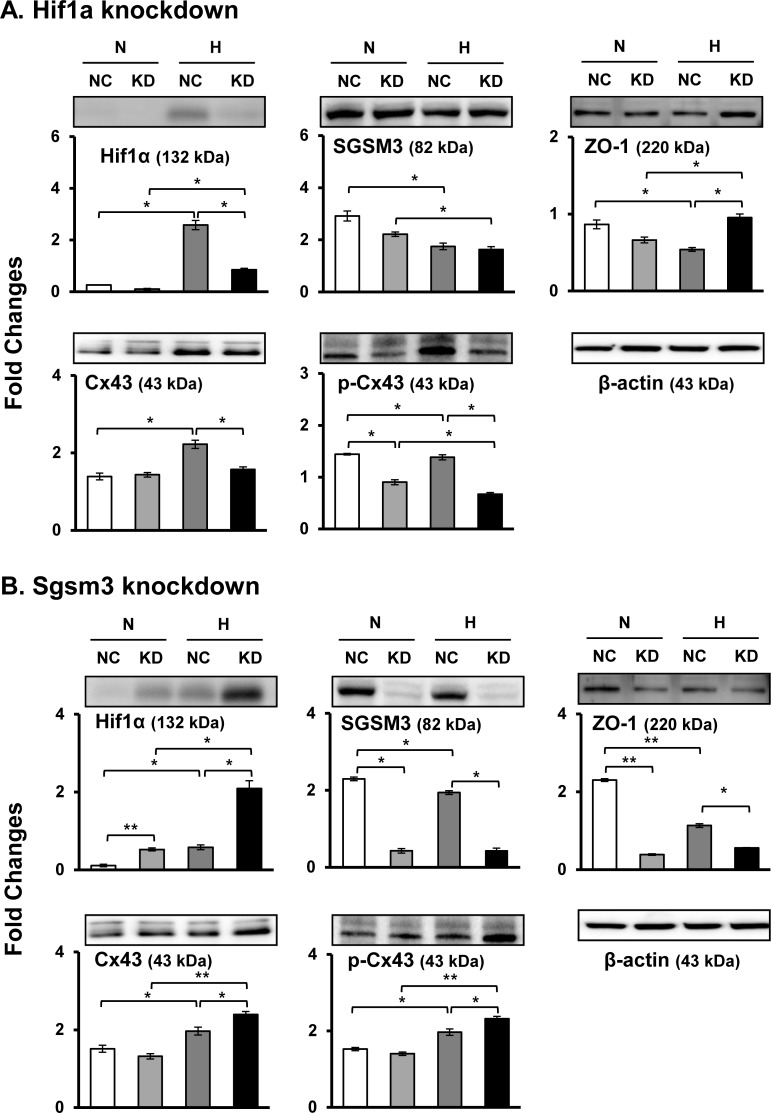
Effects of Hif1a (A) and Sgsm3 (B) knockdown on Cx43 and SGSM3 expression levels in rat MSCs under normoxic and hypoxic conditions, as measured via immunoblot analysis. Band intensity was measured as the area density and analyzed in ImageJ and relative intensity levels indicate protein levels normalized to the β-actin levels. The data are representative of two independent experiments. Significant differences between groups were determined via ANOVA, with *p* values indicated as *p<0.05 and **p<0.01. N, normoxia; H, hypoxia; NC, negative control cells; KD, knockdown cells.

For experimental proof of the interaction between SGSM3 and Cx43 and to confirm the relevance of the two proteins and Hif1α, Hif1a and Sgsm3 gene knockdown (KD) using siRNA transfection was performed in rat MSCs. After 24 h of siRNA KD, MSCs were exposed to a normoxic or hypoxic environment for 12 h. Hif1a was effectively knocked down by siRNA transfection, and Hif1a KD affected the downregulation of Cx43 expression and the upregulation of ZO-1 expression under normoxic and/or hypoxic conditions (Fig 2A). However, Hif1a KD did not change the expression of SGSM3 (Fig 2A). On the other hand, Sgsm3 was substantially knocked down by siRNA transfection, and Sgsm3 KD significantly increased the expression of Hif1α and Cx43 and attenuated the expression of ZO-1 (Fig 2B). It can be inferred that SGSM3 is closely related to Cx43, Hif1α, and ZO-1

### Effects of Sgsm3 KD on cell apoptosis under hypoxic stress

**Fig 3:**
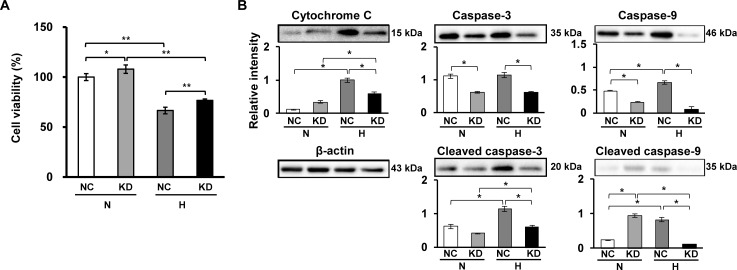
Effects of Sgsm3 knockdown on cell death and apoptosis under hypoxia in rat MSCs. Changes in cell viability (A) and apoptosis marker expression (B) in MSCs with Sgsm3 knockdown under normoxic and hypoxic conditions were measured using Ez-Cytox and immunoblot analysis, respectively. Band intensity was measured as the area density and analyzed in ImageJ and relative intensity levels indicate protein levels normalized to the β-actin levels. The data are representative of two independent experiments. Significant differences between groups were determined via ANOVA, with *p* values indicated as *p<0.05 and **p<0.01. N, normoxia; H, hypoxia; NC, negative control cells; KD, knockdown cells.

To determine the effects of Sgsm3 KD on cell death induced by hypoxic stress in rat MSCs, the viability of control and Sgsm3 KD cells was investigated under normoxic and hypoxic conditions. We found that Sgsm3 KD significantly prevented cell death induced by low oxygen under both normoxic and hypoxic conditions (Fig 3A). On the basis of these results, we hypothesized that Sgsm3 KD could induce changes in increases in apoptosis-related proteins under hypoxic stress. Therefore, apoptosis marker expression in control and Sgsm3 KD cells under different oxygen conditions was observed via immunoblot analysis. Surprisingly, Sgsm3 KD remarkably inhibited the increases in cytochrome C, caspase-3 and caspase-9 induced by hypoxic stress (Fig 3B). These results suggest that Sgsm3 KD could block hypoxia-induced apoptosis.

### Effects of Sgsm3 KD on the expression levels of cardiomyogenic factors

**Fig 4:**
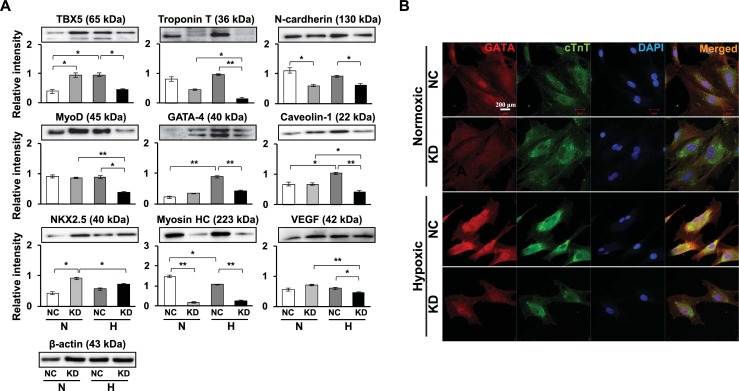
Effects of Sgsm3 knockdown on cardiogenic marker expression in rat MSCs. Changes in cardiogenic marker expression upon Sgsm3 knockdown in MSCs were measured via immunoblot analysis (A) and immunocytochemical staining (B). Band intensity was measured as the area density and analyzed in ImageJ and relative intensity levels indicate protein levels normalized to the β-actin levels (A). The nuclei were stained with DAPI (B). Scale bar = 200 μm. The data are representative of two independent experiments. Significant differences between groups were determined via ANOVA, with *p* values indicated as *p<0.05 and **p<0.01. N, normoxia; H, hypoxia; NC, negative control cells; KD, knockdown cells.

**Fig 5:**
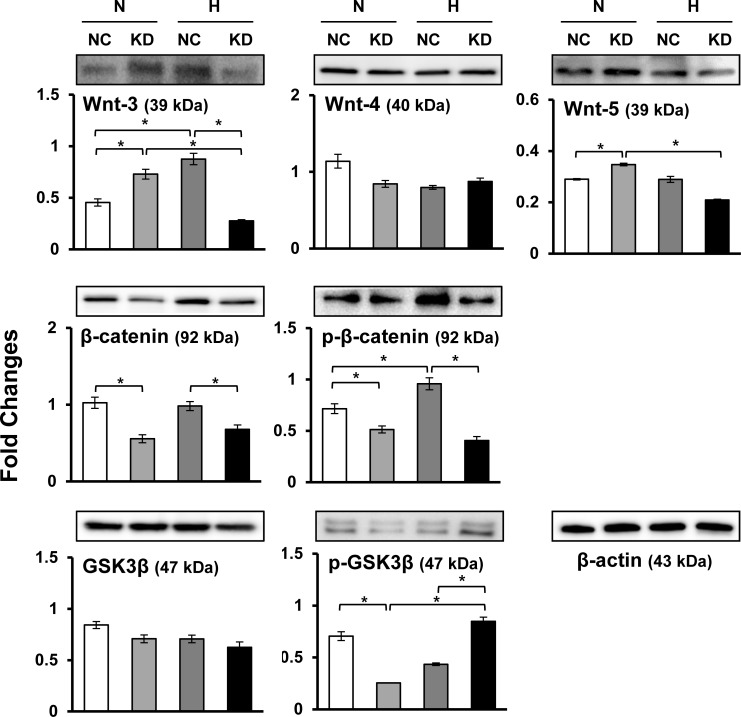
Effects of Sgsm3 knockdown on the Wnt/β-catenin pathway in rat MSCs. Changes in Wnt/β-catenin pathway-related protein expression upon Sgsm3 knockdown in MSCs were measured via immunoblot analysis. Band intensity was measured as the area density and analyzed in ImageJ and relative intensity levels indicate protein levels normalized to the β-actin levels. The data are representative of two independent experiments. Significant differences between groups were determined via ANOVA, with *p* values indicated as *p<0.05 and **p<0.01. N, normoxia; H, hypoxia; NC, negative control cells; KD, knockdown cells.

We previously found alterations in cardiomyocyte differentiation-related proteins in MSCs exposed to hypoxia [10]. Here, we investigated whether Sgsm3 KD can cause increased expression of cardiomyogenic markers in MSCs under hypoxic stress using immunoblotting and immunofluorescent staining. Consequently, Sgsm3 KD significantly decreased the expression levels of cardiomyocyte differentiation-related proteins, except NKX2.5, under hypoxia (Fig 4A). Vascular endothelial growth factor (VEGF), which promotes cardiomyocyte differentiation, was investigated in Sgsm3 KD cells exposed to hypoxia, but there was no effect of Sgsm3 KD (Fig 4A). The differential expression of cardiomyogenic factors induced by Sgsm3 KD under normoxic and hypoxic conditions was validated in MSCs using immunofluorescent staining (Fig 4B). Additionally, the effects of Sgsm3 KD on the Wnt/β-catenin signaling pathway were investigated, and decreases in Wnt-3 and β-catenin/p-β-catenin expression and increases in p-glycogen synthase kinase 3 β (GSK3β) expression were found under hypoxic conditions (Fig 5). These results imply that Sgsm3 KD could inhibit differentiation into cardiomyocytes under hypoxic stress and affect the Wnt/β-catenin signaling pathway in MSCs.

## Discussion

The SGSM family contains three members (SGSM1, SGSM2, and SGSM3) [32] and SGSMs are expressed in the central nervous system neurons and are involved in the RAP RAB family. This suggests that SGSMs play an essential part in neuronal signal transduction and vesicular transportation pathways [33]. Lan Z et al. first identified that SGSM3 interacts with Cx43 and induces its degradation, and these results suggest that SGSM3 may induce Cx43 turnover through the lysosomal pathway [34]. The Cx43 is affected by interactions between Cx43 and numerous proteins, which related to trafficking, channel construction, and degradation [35,36]. SGSM3 increases Cx43 turnover by accelerating the internalization of Cx43 from the plasma membrane [37]. On the other hand, several studies have shown that SGSM3 is associated with the risk of some cancers, including liver, breast, colorectal and bladder cancers [38–41].

We previously found that SGSM3 functions as a partner of Cx43 and inhibits Cx43 degradation in a rat MI model and cardiomyocytes [29,30]. Based on these previous data, we were curious whether SGSM3 also plays a role in cardiomyogenic differentiation in stem cells. We investigated the expression and interaction of Cx43 and SGSM3 in MSCs under low oxygen conditions, which could induce differentiation into cardiomyocytes and/or cells with comparable phenotypes in MSCs [10]. The results indicated that SGSM3 was decreased, whereas Cx43 was increased in rat MSCs under hypoxic stress. These results are consistent with the opposite expression pattern of Cx43/SGSM3 in a rat MI model and cardiomyocytes [29,30]. In addition, we found that Hif1a KD reduced Cx43 and ZO-1 expression but did not change SGSM3 expression, whereas Sgsm3 KD increased Hif1α and Cx43 expression and decreased ZO-1 expression under hypoxic conditions. These results indicate that SGSM3 is reduced by hypoxic stress in a HIF1α-independent manner and that SGSM3, HIF1α, and Cx43 are closely related; moreover, SGSM3 may induce Cx43 turnover in stem cells.

It was recently reported that connexins contribute to regulating cell growth and death. Wang D et al. investigated whether Cx43 affects MSC survival and improves therapeutic efficacy in a rat MI model [42]. The authors found that Cx43 overexpression improved cell survival and reduced infarct size in a rat MI model, indicating that Cx43 may act as a potential target for improving the therapeutic efficacy of MSCs in ischemic heart disease [42]. In addition, Cx43 expression in tongue muscle-derived stem cells was observed in the earlier stage of stem cell transplantation and contributed to less arrhythmogenicity, leading to improved survival in a mouse MI model [43]. In the present study, Sgsm3 KD ameliorated hypoxia-induced MSC death, which may be caused by Sgsm3 KD-induced increases in CX43.

MI and subsequent ischemic processes under low oxygen conditions result in extensive cardiomyocyte loss, and MSC-based therapies are gaining attention as a way to replace current techniques [44]. Although complete differentiation into functional cells has not yet been achieved, MSCs can differentiate into cardiac cell types [10,44]. In vivo studies have demonstrated that MSC-derived differentiated cells have electrophysiological functions similar to cells originating from cardiac tissue [45,46]. In addition, in vitro studies showed that MSCs have the ability to form functional gap junctions and cause voluntary rhythms [47,48]. Valiunas et al. suggested that gap junction proteins such as Cx40 and Cx43 may play a significant role in the physiology and/or pathology of cardiovascular tissues, including cardiac conduction properties and myoendothelial intercellular communication [48]. In fact, changes in Cx43 expression and distribution were shown in myocardium diseases such as hypertrophic cardiomyopathy, heart failure and ischemia [49]. In addition, Cx43 is important for maintaining late-passage MSCs during adipogenesis and regulates the osteogenic differentiation of bone marrow-derived MSCs [50,51]. These results imply that Cx43 may play a role in the cardiac differentiation of MSCs. However, to the best of our knowledge, there is no direct report about the effects of Cx43 on the cardiac differentiation of stem cells. Here, we found that Sgsm3 KD inhibits hypoxia-induced cardiac differentiation in MSCs, supporting the findings of increased Cx43 by Sgsm3 KD.

Together with the studies of the effects of Sgsm3 KD on cardiac differentiation, we investigated the influence of Sgsm3 KD on the Wnt/β-catenin pathway. Wnt/β-catenin signaling is critical in stem cell biology and is involved in cardiomyogenesis via canonical or noncanonical signaling [52,53]. β-catenin, which related to the canonical Wnt pathway, is a feature of Wnt signaling activation [54]. GSK3β is an intracellular inhibitor of the Wnt/β-catenin pathway and may block differentiation in stem cells [55]. In the current study, we found that SGSM3 KD may affect the Wnt/β-catenin pathway in a manner related to hypoxia-induced cardiogenic differentiation in MSCs. These results suggest that SGSM3 KD may attenuate cardiogenic differentiation in rat MSCs through a Wnt/β-catenin-dependent pathway.

We identified that SGSM3 interacts with Cx43 in rat MSCs under different oxygen conditions and that SGSM3 KD inhibits apoptosis and cardiomyocyte differentiation under hypoxic stress and affects the Wnt/β-catenin signaling pathway. SGSM3/Sgsm3 probably has an effect on MSC survival and has therapeutic potential in diseased hearts, but SGSM3 may worsen the development of MSC-based therapeutic approaches in regenerative medicine.
